# Genetics of Isolated Growth Hormone Deficiency

**DOI:** 10.4274/jcrpe.v2i2.52

**Published:** 2010-05-01

**Authors:** Primus E. Mullis

**Affiliations:** 1 Inselspital, Division of Paediatric Endocrinology, Diabetology&Metabolism, University Children’s Hospital, Bern, Switzerland; +41 31 632 9552+41 31 632 9550primus.mullis@insel.chPrimus E. Mullis, Division of Paediatric Endocrinology, Diabetology&Metabolism, University Children’s Hospital, Inselspital, CH−3010 Bern, Switzerland

**Keywords:** growth, human GH−gene cluster, isolated growth hormone deficiency, children

## Abstract

When a child is not following the normal, predicted growth curve, an evaluation for underlying illnesses and central nervous system abnormalities is required, and appropriate consideration should be given to genetic defects causing growth hormone (GH) deficiency (GHD). Because Insulin−like Growth Factor−I (IGF−I) plays a pivotal role, GHD could also be considered as a form of IGF−I deficiency (IGFD). Although IGFD can develop at any level of the GH−releasing hormone (GHRH)−GH−IGF axis, a differentiation should be made between GHD (absent to low GH in circulation) and IGFD (normal to high GH in circulation). The main focus of this review is on the GH gene, the various gene alterations and their possible impact on the pituitary gland. However, although transcription factors regulating the pituitary gland development may cause multiple pituitary hormone deficiency, they may present initially as GHD.

**Conflict of interest:**None declared.

## INTRODUCTION

Historically, over the last decades, growth disorders were managed on the basis of a growth hormone (GH)−oriented classification system. However, nowadays we are well aware that: a) GH is not the major mediator of skeletal growth; b) scepticism as well as criticisms are adequate and accepted while analyzing the variable results of the various GH stimulation tests; c) many genetic defects have been described and, therefore, have presented important insights into the molecular basis of also non−GH deficient growth failure.

Therefore, when a child is not following the normal, predicted growth curve, an evaluation for underlying illness and central nervous system abnormalities is required. Where appropriate, genetic defects causing GH deficiency (GHD) should be considered. Because Insulin−like Growth Factor−I (IGF−I) plays a pivotal role in growth, where it mediates most, if not all, of the effects of GH, in fact GHD could also be considered somehow as secondary IGF−I deficiency (IGFD). Although IGFD can develop at any level of the GH−releasing hormone (GHRH)−GH−IGF axis, we would like to differentiate, however, between GHD (absent to low GH in circulation) and IGFD (normal to high GH in circulation). The main focus of this review is on the GH gene cluster, the GHRH− as well as the GHRH−receptor− gene.

## CLASSIFICATION OF ISOLATED GROWTH HORMONE DEFICIENCY

**Structure and Function of GH and CS Genes**

The GH gene cluster consists of five structurally similar genes in the order 5' [GH−1, CSHP (chorionic somatomammotropin pseudogene), CSH−1 (chorionic somatomammotropin gene), GH−2, CSH−2] 3' encompassing a distance of about 65,000 bp (65 kb) on the long arm of chromosome 17 at bands q22−24(1). The GH−1 gene encodes the mature human GH, a 191−amino acid (aa) peptide, and consists of five exons and four introns (1, 2, 3). Approximately 75% of circulating GH is expressed in the anterior pituitary gland as a major 22−kDa product, whereas alternative splicing can give rise to minor forms (2, 3, 4). The most prominent minor form (5−10%) is a bioactive 20−kDa GH peptide isoform that results from the use of a cryptic 3’ splice site in E3, deleting aa 32−46 (4, 5, 6, 7). The GH−2 gene encodes a protein (GH−V) that is expressed in the placenta rather than in the pituitary gland and differs from the primary sequence of GH−N (product of GH−1 gene) by 13 aa. This hormone replaces pituitary GH in the maternal circulation during the second half of pregnancy. The CSH−1, CSH−2 genes encode proteins of identical sequences, whereas the CSHP encodes a protein that differs by 13 aa and contains a mutation (donor splice site of its second intron) that should alter its pattern of mRNA splicing and, therefore, the primary sequence of the resulting protein. The extensive homology (92−98%) between the immediate flanking, intervening, and coding sequences of these 5 genes suggests that this multigene family arose through a series of duplicational events. With the exception of CSHP, each gene encodes a 217 aa pre−hormone that is cleaved to yield a mature hormone with 191−aa and a molecular weight of 22kDa. The expression of GH−1 gene is further controlled by cis− and trans−acting elements and −factors, respectively (2, 3, 4, 5, 6, 7, 8).

**Familial Isolated GHD**

Short stature associated with GHD has been estimated to occur in about 1/4,000 − 1/10,000 in various studies (9, 10, 11). While most cases are sporadic and are believed to result from environmental cerebral insults or developmental anomalies, 3−30% of cases have an affected first−degree relative suggesting a genetic aetiology. Since magnetic resonance examinations detect only about 12−20% anomalies of either hypothalamus or pituitary gland in isolated GHD (IGHD), it can be assumed that many genetic defects may not be diagnosed and a significantly higher proportion of sporadic cases may have indeed a genetic cause (12). Familial IGHD, however, is associated with at least four Mendelian disorders (2, 3, 4, 5, 6, 7, 8), including two forms that have autosomal recessive inheritance (IGHD type IA, IB) as well as autosomal dominant (IGHD type II) and X−linked (IGHD III) forms. Table 1 depicts the mutational spectrum of GHD, which is discussed in greater detail later in the review.

**IGHD Type IA**

In 1970, IGHD type IA was first described by Ruth Illig in three Swiss children with unusually severe growth impairment and apparent deficiency of GH (13). Affected individuals occasionally have short length at birth and hypoglycaemia in infancy, but uniformly develop severe growth retardation by the age of six months. Their initial good response to exogenous GH is hampered by the development of anti−GH−antibodies leading to dramatic slowing of growth (2, 14).

**GH−1 Gene Deletions**

In 1981, Phillips et al (14) examined the genomic DNA from these Swiss children and discovered, using Southern blotting technique that the GH−1 gene was missing. Subsequently, additional cases of GH−1 gene deletions have been described responding well to the GH treatment. The development of anti−GH antibodies is an inconsistent finding in IGHD IA patients despite the presence of identical molecular defects (homozygosity for GH−1 gene deletions) (15). The frequency of GH−1 gene deletions as a cause of GHD varies among different populations and according to the chosen criteria and definition of short

stature (1). The sizes of the deletions are heterogeneous with the most frequent (70−80%) being 6.7kb (2, 8). The remaining deletions described include 7.6, 7.0, 45 kb, as well as double deletions within the GH gene cluster (2, 8). At the molecular level, these deletions involve unequal recombination and crossing over within the GH−gene cluster at meiosis (2).

**GH−1 Gene Frameshift− and Nonsense Mutations**

Single−base pair deletions and nonsense mutations of the signal peptide may result in an absent production of mature GH and in the production of anti−GH−antibodies on exogenous replacement therapy (8, 16, 17, 18, 19).

**IGHD Type IB**

Patients with IGHD type IB are characterized by low but detectable levels of GH (<7 mU/l; <2.5 ng/ml), short stature (<−2 SDS for age and sex), growth deceleration and height velocity less than 25th percentile for age and sex, significantly delayed bone age, an autosomal recessive inheritance (two parents of normal height; two sibs affected), no demonstrable direct and/or endocrine cause for IGHD, and a positive response and immunological tolerance to treatment with exogenous GH. This subgroup of IGHD has been broadened and reclassified on the basis of the nature of their GH gene defects and includes splice site mutations of the GH gene, even an apparent lack of GH has been found by RIA. The phenotype of IGHD type IB, therefore, is more variable than IA. In one family, the children may resemble IGHD type IA, whereas in other families, growth during infancy is relatively normal and growth failure is not noted until mid−childhood. Similarly, GH may be nearly lacking or simply low following stimulation test. This heterogeneous phenotype suggests that there is more than one candidate gene causing the disorder, as summarized recently.

**Candidate Genes in IGHD Type IB**

Some of the components of the GH pathway are unique to GH (18, 20), whereas many others are shared. In patients with IGHD, mutational changes in genes specific to the GHRH−GH axis are of importance and there is a need to focus on them.

**GHRH−Gene**

Many laboratories put a lot of energy to define any GHRH gene alterations. To date, no GHRH gene mutations or deletions causing IGHD have been reported (8, 21, 22). This is, however, somewhat a surprising observation, and the GHRH gene must still be considered a candidate gene for familial forms of IGHD.

**GHRH−Receptor (GHRHR) Gene**

In 1992, Kelly Mayo cloned and sequenced the rat and human GHRH−receptor (GHRHR) gene that provided the opportunity to examine the role of GHRHR in growth abnormalities that involve the GH−axis (23). Sequencing of the GHRHR gene in the little−mouse (lit/lit) showed a single nucleotide substitution in codon 60 that changed aspartic acid to glycine (D60G) eliminating the binding of GHRH to its own receptor (24). As the phenotype of IGHD type IB in humans has much in common with the phenotype of homozygous lit/lit mice including autosomal recessive inheritance, time of onset of growth retardation, diminished secretion of GH and IGF−I, proportional reduction in weight and skeletal size, and delay in sexual maturation, the GHRHR gene was searched for alteration in these patients suffering from IGHD type IB (25, 26). Wajnrajch et al (26) reported a nonsense mutation similar to the little mouse in an Indian Muslim kindred. Furthermore, in two villages in the Sindh area of Pakistan, Baumann (27) reported another form of severe short stature caused by a point mutation in the GHRHR gene resulting in a truncation of the extracellular domain of this receptor. Individuals who are homozygous for this mutation are very short (−7.4 SDS) but normally proportioned. They appear to be of normal intelligence, and at least some are fertile. Biochemical testing revealed that they have normal levels of GHRH and GH binding protein (GHBP), but undetectable levels of GH and extremely low levels of IGF−I. Later, families from Sri Lanka, Brazil, United States, Spain as well as Pakistan were reported (28, 29, 30, 31). Mutations in the GHRHR gene have been described as the basis for a syndrome characterized by autosomal recessive IGHD and anterior pituitary hypoplasia, defined as pituitary height more than 2 SD below age−adjusted normal, which is likely due to depletion of the somatotrop cells (OMIM: 139190). In a most recent report, however, certain variability in anterior pituitary size even in siblings with the same mutation was described (32). This finding is of importance, as it was thought that patients with a GHRHR gene defect invariably have an anterior pituitary gland hypoplasia and that GHRHR gene mutations can be excluded in the absence of this pathological feature, because GHRHR may be critical for pituitary gland development and function of the somatotropes (33, 34). Further, Hilal et al (35) discussed most interestingly the possible role of GHRHR in the proper development of extrapituitary structures, through a mechanism that could be direct or secondary to severe GHD.

Overall, mutations in the human GHRHR gene can impair ligand binding and signal transduction, and have been estimated to cause about 10% of autosomal recessive familial IGHD (36). Mutations reported to date include six splice donor site mutations, two microdeletions, two nonsense mutations, seven missense mutations, and one mutation in the promoter (35, 36). These mutations have an autosomal recessive mode of inheritance, and heterozygous individuals do not show signs of IGHD, although the presence of an intermediate phenotype has been hypothesized. Conversely, patients with biallelic mutations have low serum IGF−1 and GH levels (with absent or reduced GH response to exogenous stimuli), resulting − if not treated− in proportionate dwarfism (36, 37).

**Muscarinic Acetylcholine Receptor (mAchR)**

Acetylcholine, as a neurotransmitter, exerts many of its actions via interaction with one or more of the five mammalian muscarinic acetylcholine receptor (mAchR) subtypes, M1−M5. The importance of cholinergic pathways in the regulation of GH secretion in humans is well established. Central cholinergic stimulation gives rise to an increase in GH release, whereas cholinergic blockade is followed by a blunting in GH secretion (38). Acetylcholinesterase inhibitors, which indirectly activate cholinergic neurotransmission, are believed to act by reducing the release of somatostatin (SRIF), thus increasing spontaneous GH secretion, and potentiating GH responses to GHRH or to other stimuli. Conversely, muscarinic cholinergic receptor antagonist drugs reduce spontaneous GH release as well as GH responses to GHRH, sleep, exercise, L−dopa, glucagon, arginine, and clonidine. Mouse models have been generated, in which a specific subtype of mAchR has been ablated by genetic engineering (39). These animals have a wide variety of phenotypic abnormalities but not growth failure, seemingly showing, that at least in rodents, the lack of muscarinic receptor function would not cause a significant reduction in GH secretion. However, very recently a murine model was created, in which the function of the M3 receptor was ablated in both alleles exclusively in the central nervous system (40). In this model, body length is reduced, and this is associated with significantly reduced GH and IGF−I serum levels and a reduction in pituitary somatotroph cell mass. Although the degree of growth retardation and pituitary hypoplasia is not as marked, the phenotype of this animal has a striking similarity with the murine model of ablation of the GHRH gene (41), and with the naturally occurring mutation in the GHRHR gene that occurs in the little mouse (24). These observations are consistent with the hypothesis that the neuronal muscarinic receptors play an important role in controlling GH secretion. Based on all the above observations, we hypothesized that a subgroup of IGHD type IB families may have inactivating mutations in these receptors. To test this hypothesis, we analyzed the M1−M5 receptor genes in 39 of these families.

However, we concluded from this study that mAchR mutations are absent or rare (less than 2.6%) in familial IGHD type IB (42).

**Ghrelin Receptor, GH Secretagogue Receptor (GHSR)**

To date, there is one recent report describing a loss of function of the constitutive activity of the GH secretagogue receptor (GHSR) in familial short stature (43, 44). GHSR is highly expressed in the brain and in the pituitary gland. The first endogenous ligand of this receptor was discovered back in 1999 and was named ghrelin (45). Although pharmacological studies have demonstrated that this endogenous ligand stimulates, through the GHSR, GH secretion and appetite, the physiological importance of the GHSR−dependent pathways remains an open question that gives rise to much controversy (43).

**Homeobox Gene Expressed in ES Cells; HESX1**

It has been shown that familial septo−optic dysplasia (SOD), a syndromic form of congenital hypopituitarism involving optic nerve hypoplasia and agenesis of midline brain structures, may be associated with homozygosity for an inactivating mutation in the homeobox gene hesx1/HESX1. Importantly, a small proportion of mice heterozygous for the hesx1 null allele show a milder form of SOD, implying that heterozygosity in human HESX1 gene alteration may lead to a mild phenotype of IGHD only (46). Therefore, actually the HESX1 gene has to be studied whenever looking for any molecular reason causing IGHD type IB (47).

**SOX3 SRY (Sex Determining Region Y)−Box 3**

SOX3 is located on the X−chromosome and both under− and overdosages of the gene lead to hypopituitarism (48, 49). Male patients present with variable hypopituitarism (combined pituitary hormone deficiency (CPHD) or IGHD) and infundibular hypoplasia, an ectopic/undescended posterior pituitary and abnormalities of the corpus callosum with or without mental retardation, in other words, this gene needs a closer look as well while studying IGHD (47, 50).

**Specific Trans−Acting Factor to GH−Gene**

Any alteration to the specific transcriptional regulation of the GH−1 gene may produce IGHD type IB. Mullis et al (51) have reported a heterozygous 211 base pairs (bp) deletion within the retinoic acid receptor a gene causing the phenotype of IGHD type IB.

**Transcription Factors, Important for Pituitary Gland Development**

In addition, as the occurrence of the various hormonal deficiencies caused by transcription factors important for the pituitary gland development may vary quite drastically also with a family presenting with an identical gene defect, GHD can be the only defect at the beginning. Therefore, the two most important transcription factors, namely POU1F1 (Pit−1) and PROP 1, are shortly discussed (Table 2).

**POU1F1 (PIT1)**

The pituitary transcription factor PIT−1 is a member of the POU−family of homeoproteins, which regulates important differentiating steps during embryological development of the pituitary gland as well as target gene function within the postnatal life (8). Further, it is 291 aa in length, contains a transactivation domain and two conserved DNA−binding domains: the POU−homeodomain and the POU−specific domain. As PIT1 is confined to the nuclei of somatotropes, lactotrops and thyrotropes in the anterior pituitary gland, the target genes of PIT1 include the GH−, prolactin− (PRL) and the thyrotropin (TSH)−subunit−, and the POU1F1 gene itself. Therefore, the defects in the human POU1F1 gene known so far have all resulted in a total deficiency of GH and PRL, whereas a variable hypothyroidism due to an insufficient TSH secretion, at least during childhood, has been described (Table 2). Although it is important to stress that the clinical variability is due to other factors than the exact location of the mutation reported, the type of inheritance, however, seems to correlate well with the genotype. The first mutation within the POU1F1 gene was identified by Tatsumi (52). The majority of the mutations reported so far are recessive, however, a number of heterozygous point mutations have been reported (53). Of those, the aa substitution R271W (Arg271Trp) seems to be a “hotspot”. Further, the dominant negative effect of the R271W POU1F1 form has been recently challenged by Kishimoto et al (54). Although most cases with R271W were sporadic and presenting with an autosomal dominant mode of inheritance, Okamoto et al (55) reported a family with normal family members, who were clearly heterozygous for that mutation. Therefore, further in vitro expression studies were performed that could not confirm its dominant negative effect, which is well in contrast with the original report using identical experimental conditions (8, 54).

**PROP1**

Wu et al (56) described four families, in which CPHD was associated with homozygosity or compound heterozygosity for inactivating mutations of the PROP1 gene. PROP1 (prophet of Pit1) is a paired−like homeodomain transcription factor and, originally, a mutation in this gene (Ser83Pro) was found causing the Ames dwarf (df) mouse phenotype (57). In mice, Prop1 gene mutation primarily causes GH, PRL and TSH deficiency, and in humans, PROP1 gene defects also appear to be a major cause of CPHD. In agreement with the model of Prop1 playing a role in commitment of dorsal lineages (GH, PRL and TSH), Prop1 mutant mice exhibit a dorsal expansion of gonadotrophs that normally arise on the ventral side.

To date, many different missense, frameshift and splice site mutations, deletions, insertion have been reported and it has been realized that the clinical phenotype varied not only among the different gene mutations, but also among the affected siblings with the same mutation (58, 59). In addition, although the occurrence of the hormonal deficiency varies from patient to patient (8), the affected patients as adults were not only GH, PRL and TSH deficient, but also gonadotropin deficient (Table 2). The three tandem repeats of the dinucleotides GA at location 296−302 in the PROP1 gene represent a “hot−spot” for CPHD (58, 59, 60). Low levels of cortisol have also been described in some patients with PROP1 gene mutations (61). In addition, pituitary enlargement with subsequent involution has been reported in patients with PROP1 mutations (61). The mechanism, however, underlying this phenomenon remains still unknown.

**IGHD Type II**

Focusing on the autosomal dominant form of IGHD, type II (IGHD II) is mainly caused by mutations within the first six bp of intervening sequences 3 (5’IVS−3) (8, 62, 63, 64, 65, 66), which result in a missplicing at the mRNA level and the subsequent loss of E3, producing a 17.5−kDa hGH isoform (8, 65). This GH product lacks aa 32−71 (del32−71 GH), which is the entire loop that connects helix 1 and helix 2 in the tertiary structure of hGH (67, 68). Skipping of E3 caused by GH−1 gene alterations other than those at the donor splice site in 5’IVS−3 has also been reported in other patients with IGHD II. These include mutations in exon 3 (E3) splice enhancer ESE1 (E3+1G−>T:ESE1m1; E3+2A−>C:ESE1m2, E3+5A−>G:ESE1m3) as well as ESE2 (downstream of the cryptic splice site in E3; ESE2: Δ721−735) and within

suggested intron splice enhancers (ISE) (IVS−3+28 G−>A: ISEm1; IVS−3del+28−45: ISEm2) sequences (8, 64, 69, 70, 71, 72, 73, 74, 75). Such mutations lie within purine−rich sequences and cause increased levels of E3 skipped transcripts (64, 69, 70, 71, 73, 74, 75), suggesting that the usage of the normal splicing elements (ESE1 at the 5’ end of E3 as well as ISE in intron 3) may be disrupted (73, 74, 75). Importantly, the first 7 nucleotides in E3 (ESE1) are crucial for the splicing of GH mRNA (75) such that some nonsense mutations might cause skipping of one or even more exons during mRNA splicing in the nucleus. This phenomenon is called nonsense−mediated altered splicing (NAS); its underlying mechanisms are still unknown (76). Furthermore, there is a recent report of Vivenza et al (77) presenting a patient with a specific deletion within intron 3 leading to E3 skipping, which underlines the importance of intron length on the splicing machinery, as it was previously suggested by the elegant work by Ryther et al (75). In addition to the above described splice site mutations that result in the production of del32−71 GH, three other mutations within the GH−1 gene (missense mutations) are reported to be responsible for IGHD II, namely, the substitution of leucine for proline, histidine for arginine and phenylalanine for valine at aa positions 89 (P89V), 183 (R183H) and 110 (V110F), respectively (66, 78, 79).

At the functional level, the 17.5−kDa isoform exhibits a dominant−negative effect on the secretion of the 22−kDa isoforms in both tissue cultures as well as in transgenic animals (80, 81, 82). The 17.5−kDa isoform is initially retained in the endoplasmic reticulum, disrupts the Golgi apparatus, impairs both GH and other hormonal trafficking (83), and partially reduces the stability of the 22−kDa isoform (80). Furthermore, transgenic mice overexpressing the 17.5−kDa isoform exhibit a defect in the maturation of GH secretory vesicles and the anterior pituitary gland is hypoplastic due to a loss of the majority of somatotropes (73,80,81). Trace amounts of the 17.5−kDa isoforms, however, are normally present in children and adults of normal growth and stature (84), and heterozygosity for A731G mutation (K41R) within the newly defined ESE2 (which is important for E3 inclusion) led to approximately 20% E3 skipping resulting in both normal as well as short stature (73, 75, 85).

From the clinical point of view, severe short stature (<−4.5 SDS) is not present in all affected individuals, indicating that in some forms of IGHD II, growth failure is less severe than one might expect (66). It has been hypothesized that children with splice site mutations may be younger and shorter at diagnosis than their counterparts with missense mutations (66). In addition, more recent in vitro and animal data suggest that both a quantitative and qualitative difference in phenotype may result from variable splice site mutations causing differing degrees of E3 skipping (8, 85, 86, 87, 88, 89). In summary, these data suggest that the variable phenotype of autosomal dominant GHD may reflect a threshold and a dose dependency effect of the amount of 17.5−kDa relative to 22−kDa hGH (81, 82, 85). Specifically, this has a variable impact on pituitary size, as well as on onset and severity of GHD and, unexpectedly, the most severe, rapid onset forms of GHD might be subsequently associated with the evolution of other pituitary hormone deficiencies (90, 91).

**IGHD Type III**

This reported type is X−linked, recessively inherited. In these families, the affected males were immunoglobulin−as well as GH−deficient (92, 93). Recent studies have shown that the long arm of X−chromosome may be involved and that the disorder may be caused by mutations and/or deletions of a portion of the X−chromosome containing two loci, one necessary for normal immunoglobulin production, and the other for GH expression (94). In addition, Duriez et al (95) reported an exon−skipping mutation in the btk−gene of a patient with X−linked agammaglobulinemia and IGHD.

**Table 1:**
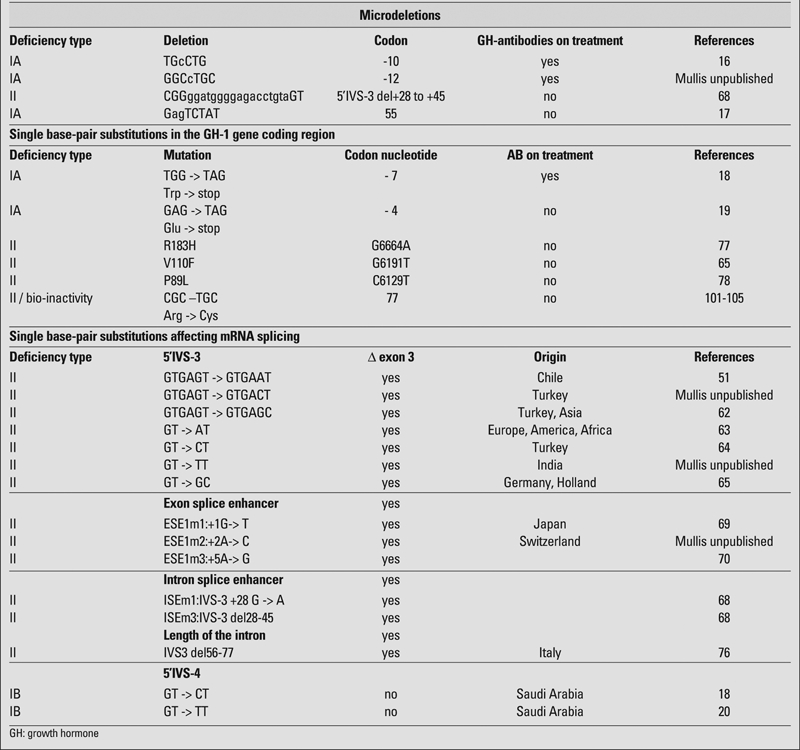
Mutational spectrum of GH−deficiency

**Table 2:**
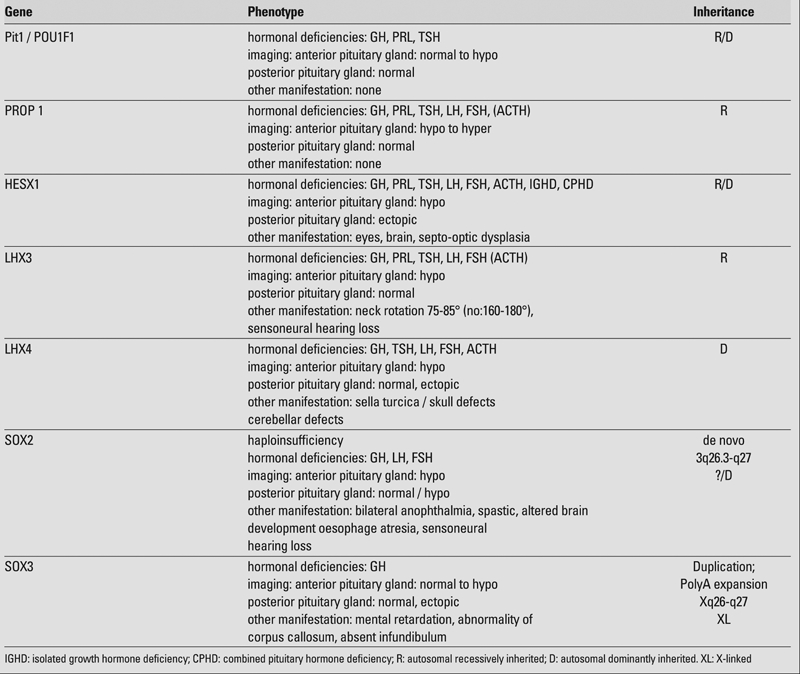
Transcription factors of clinical importance

## BIOINACTIVE GH

Short stature associated with bioinactive GH was first suggested and described by Kowarski and co−workers in 1978 (96). It is clinically characterized by normal or slightly increased GH secretion, pathologically low IGF−I levels, and normal catch−up growth on GH−replacement therapy. On a clinical basis, additional cases of bioinactive GH were described in the eighties (97, 98, 99, 100, 101). Chihara and co−workers (102, 103, 104) reported two missense mutations (R77C and D112G) in the GH−1 gene leading to Kowarski’s syndrome in two Japanese patients. However, these mutations were both found in the heterozygous state only, and furthermore, the mutation R77C was also identified in the normal−statured father. Further, six GH variants were suggested to be bioinactive by Millar and co−workers (85). Again, all these mutations were found in the heterozygous state and no clear correlation between laboratory/clinical phenotype and patient genotype was shown. Later, also our group described a heterozygous R77C mutation in the GH molecule in a patient with growth retardation and delayed pubertal development. However, no differences between wt−GH and GH−R77C were found by functional characterization of the GH−R77C through GHR binding, activation of JAK2/STAT5 pathway and additional secretion studies together with cell proliferation when stably GHR transfected cells (293GHR) were used (105, 106). On the other hand, reduced capability of GH−R77C to directly induce GHR/GHBP gene transcription rate could indirectly affect the levels of GHBP in the circulation of our patient. In addition, this group of patients deserves further attention, because they could represent a distinct clinical entity underlining that an altered GH peptide may cause partial GH insensitivity through direct impact on GHR/GHBP gene expression leading to the delay of growth and pubertal development. Finally, GH−R77C is not invariably associated with short stature, although the serum IGF−I levels are low, the GH is elevated, and the GHBP levels are somewhat low, consistent with some degree of GH insensitivity, which is, presumably, compensated for by excess of GH production. Whether this is due to GH receptor transcription defects, remains unclear.

Furthermore, in one of the more convincing cases of bioinactive GH reported to date, a homozygous missense mutation (bp:G705C; aa:C53S) leading to disruption of the disulfide bond between Cys−53 and Cys−165 was found in a short (−3.6SDS) Serbian boy. Both GHR binding as well as JAK2/STAT5 signalling activities were markedly reduced (107).

## FUNCTIONAL ANALYSIS OF ANY GENE VARIANT IS IMPORTANT

To make the story even more complicated, we reported a patient suffering from a specific form of IGHD II caused by a GH−1 gene alteration on a hypomorphic partial agonistic allele, emphasizing the importance of detailed functional analysis of GH variants. The patient was heterozygous for the GH−R178H mutation (108). Clinical findings combined with the experimental data of secretion studies confirmed the diagnosis of IGHD II. However, although the GH concentration after stimulation was reduced, admittedly supporting the diagnosis of GHD, neither the severity of short stature (−6.0 SDS at the chronological age of 5 years) nor the low IGF−1 concentrations could be fully explained. Therefore, further functional characterization of this GH mutant was performed through studies of GHR binding and activation of the JAK2/STAT5 pathway. Binding activity and the bioactivity of GH−R178H were investigated and compared with the wt−GH and revealed that GH−R178H by itself behaves more like a partial agonist. Therefore, phenotype and hormonal data underlined the fact that GH−R178H mutation expressed from a hypomorphic partial agonistic allele seems to functionally overlie IGHD II in our patient (108).
